# Professional Etiquette

**Published:** 1882-12

**Authors:** 


### ARTICLE III.

### *Professional Etiquette.*

An editorial in the *Med. and Surgical Reporter* (Nov. 4, 1882) treats ot this subject in such a commendable manner that we publish it for the benefit of the dental profession:

"We fear this is one of the lost arts among many of our profession. What it means, what are its mandates, what it obliges us to do, no one can plead ignorance of, simply because every decent and respectable man has within his innermost self that little monitor constantly advising him to do unto others as he would that they should do unto him. This, after all, is the foundation of etiquette and of justice in every profession and in every walk of life. There are some men whose moral sense seems so little developed that they fail to realize when they transgress this golden rule, and for such, one can but have pity, and charitably say, forgive them, for they know not what they do. But the majority of our profession have sufficient intelligence, education and innate refinement to cause them to thoroughly appreciate their own littleness and meanness when they degrade themselves to try and secure their own preferment by belittling those whom they feel are too noble and too much above their dirty level to meet them with their own soiled weapons. Feeling this, many conscienceless men are ever ready to aggressively and remorselessly assail others, and to, so as to speak, stab them in the dark, trustin that they will never recognize their supposed disguised and ought to be despised assailants.

There is another class of professional parasites, who, while they do not desire to injure their brethern, yet are so constituted, and possess such a small mental calibre, that they cannot resist the temptation to, vulture-like, pounce down upon an imagined weakness or failing in their confrères, and by distorting and magnifying it into gigantic proportions, hope to raise themselves by the supposed thrust downward they have given to some worthy and innocent man. There are so many open questions in medicine, and so many important questions upon which men of equally great eminence entertain directly divergent views, that it is not difficult for one so disposed to criticise almost any statement that may be made or any action that may be performed, and to cite good authority to support his criticism, lacking, the while, the candor or honesty to admit that he whom he criticises can produce equally strong authority for his statements. Such miserably mean men always come to grief in the long run, for be it said, to the credit of human nature, man, on the whole, realizes that honesty is the best policy; and though water for a time may be forced up grade, yet when left to itself must, according to nature's inevitable laws, seek its level; so these men are ultimately detected in their nefarious attempts at self-advancement, and are forced, by public and private opinion, to occupy that low level to which all kinds of dregs, animal and vegetable, naturally belong.

So, then, it is pleasing to the honesty of every right-minded man when such despicable men, (for we can call them by no more gentle name) are exposed. Such has been recently done in England, in the case of two professional back-biters. In the one case a surgeon was engaged to attend a case of compound fracture. When the man was discharged, cured, the physician presented a bill for sixty dollars. Instead of paying it, the patient *acting under the advice of another physician*, instituted a suit for damages, on the ground of malpractice, basing his claim on "forgetfulness or ignorance of all the rules of art, and in particular on the employment of perchloride of iron to arrest hemorrhage." A committee of experts, composed of medical men, appointed by the court, concluded in favor of the surgeon; the man offered to pay the bill and withdraw his suit; to this the surgeon objected, when it was ruled by the court that the man should pay the original bill, all costs and one hundred dollars damages to the surgeon. In the second case both plaintiff and defendant were medical men. An illiterate man was sent to a certain city, with a card from a doctor in a neighboring town, recommending him to the care of Dr. A. An omnibus driver, by mistake, took him to the house of Dr. B, who kept both card and patient. Dr. A heard of the occurrence, and meeting Dr. B on the street, upbraided him with his unprofessional conduct, and, called him offensive names. B, seeing that his position was compromised, summoned A before the courts, where the tables were entirely turned; B, was condemned to pay A $1000, damages, and an appeal resulted in a confirmation of the judgment.

Such cases should delight every honest man and every reputable practitioner should constitute himself a detective to bring to the bar of justice and purge from the list and companionship of respectable physicians, all who endeavor to advance themselves by libeling honest and capable members of our profession.

It is hard enough for a man to succeed in this world of struggling, even though he receives fair treatment from all, but when the miserable back-biter comes in to injure him his lot is truly deplorable. So then let us have cone with unseemingly contention in our great profession."

